# Behavioural Welfare Implications of Human Handling Practices in Zoo Snakes

**DOI:** 10.1002/vms3.71218

**Published:** 2026-09-14

**Authors:** Chelsea Kennedy, Sara L. Hanson, Connor Walker, Eduardo J. Fernandez

**Affiliations:** ^1^ School of Animal and Veterinary Sciences Adelaide University Roseworthy SA Australia

**Keywords:** animal–visitor interactions, behavioural observations, handling practices, reptile behaviour, snake welfare

## Abstract

**Background:**

Handling reptiles, particularly snakes, is common in ambassador–animal and animal–visitor interaction programmes. However, relatively little is known about the behavioural and welfare impacts of these interactions on snakes.

**Objectives:**

This study examined whether staff‐led handling during visitor interactions impacted snake behaviour by comparing before and after handling and between handling and non‐handling days.

**Methods:**

Observations were conducted at Cleland Wildlife Park (South Australia, Australia) on three individual snakes: one woma python (*Aspidites ramsayi*) and two Children's pythons (*Antaresia childreni*). Behavioural data were collected using cameras in ZooMonitor using focal pinpoint sampling and an adapted reptile‐based ethogram. Sixteen behaviours were categorised into five behavioural classes: Active, Inactive, Abnormal, Out of sight, and Other. All footage was scored blind to handling conditions.

**Results:**

No significant differences were detected in active or abnormal behaviour before versus after handling. However, inactive behaviour increased and out of sight behaviour decreased following handling. When handling days were compared with non‐handling days, abnormal behaviour was significantly lower on handling days, while no significant differences were detected for behaviours classified as Other.

**Conclusions:**

Short‐term, staff‐led handling during animal–visitor interactions was not associated with substantial changes in snake behaviour. The observed increase in inactive behaviour and decrease in out of sight behaviour following handling may reflect minor short‐term behavioural adjustments to human interaction rather than pronounced welfare effects. Overall, these findings suggest that, under the conditions examined, brief handling had limited direct behavioural impacts on the snakes studied.

**Highlights:**

- Although snakes are commonly displayed in zoos, little is known about proper behavioural welfare for the taxa.
- Little is also known about the impact of visitor interactions on snakes.
- We examined the impact of a snake handling interaction on three zoo‐housed snakes.
- Abnormal behavior was lower on handling days while visibility increased following handling, suggesting a neutral to positive welfare effect.
- Future research should focus on better understanding proper welfare metrics for snakes.

## Graphical abstract

This study examined behavioural responses to staff‐led handling in three zoo snakes. Handling increased inactive behaviour and reduced time out of sight after interactions, while abnormal behaviour was lower on handling days. Overall, short‐term handling produced few substantial behavioural changes, suggesting minimal welfare impacts in these regularly handled snakes.

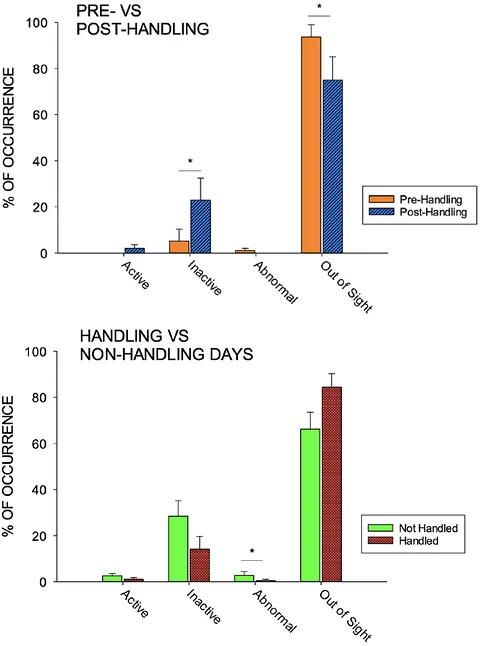

## 1 Introduction

Animal interaction programs (AIPs), a type of animal–visitor interaction (AVI), are a popular experience offered by zoos and wildlife parks, aiming to engage visitors, educate them, and raise awareness about animal behaviour and conservation (Cooper‐Rogers et al. 2026; Fischer et al. 2024; Sherwen and Fernandez 2024). Snakes are frequently the choice of reptile utilised for visitor interactions due to their unique biology and their ability to challenge perceptions and assumptions of the public about their demeanour (Fulkerson 2025; Rank et al. 2021). Over the past few decades, the popularity of interactive programmes has increased; however, there is limited research relating to visitor handling and its implications for the captive snakes’ welfare (Cooper‐Rogers et al. 2026; D'Cruze et al. 2019; Huo et al. 2025). This knowledge gap poses a problem for the welfare of captive snakes, not only in zoos and wildlife parks but also as pets, with reptiles often overlooked in favour of more traditionally studied animals such as mammals and birds (Binding et al. 2020; Burghardt 2017).

Although limited existing research on the welfare and behaviour of captive snakes does exist, it predominantly focuses on enclosure enrichment, complexity, and physical health (Hanson et al. 2025). For instance, studies have demonstrated that increasing enclosure complexity, among other environmental changes, is associated with greater behavioural diversity and reduced stress‐related behaviours in snakes. However, these findings may not directly extend to handling (Almli and Burghardt 2006; Hoehfurtner et al. 2021; Nagabaskaran et al. 2021; Spain et al. 2020).

Whereas previous research has shown that enrichment is a critical component of captive snake welfare, direct animal–visitor handling practices remain poorly examined (Hoehfurtner et al. 2021; Williams et al., 2023). This knowledge gap is concerning, considering that animal–visitor handling practices with snakes often involve direct contact and repeated interaction. This could lead to different welfare outcomes, positive or negative, that do not compare to passive enclosure modification. As noted, the existing research conducted on the behaviour of captive snakes is limited in terms of visitor interaction handling. However, research into validated indicators of stress in snakes has steadily increased in recent years; behavioural displays, which could include tongue flicking, boundary contact, defensive postures, striking, and excessively increased or decreased locomotion, have previously been suggested (Chiszar et al. 1980; Huo et al. 2025; Warwick 2023; Warwick et al. 2013). Nonetheless, these require further experimental validation to be confidently employed as welfare measures by zoos and wildlife parks. With the widespread and increased use of snakes in direct AIPs, the absence of research on the topic represents both a scientific and welfare concern (Cooper‐Rogers et al. 2026; Fulkerson 2025).

This experimental study aimed to address the gap in knowledge regarding the potential behavioural impacts of direct handling on captive snakes. By using video monitoring and a standardised snake behavioural ethogram, the study compared the behaviour of snakes on days when they were handled with days when they were not handled, as well as pre‐ and post‐handling effects. It was hypothesised that if handling was a negative experience for the snakes, those that participated in regular human handling would exhibit a greater prevalence of stress‐related behaviours on handling days compared to non‐handling days. Likewise, if handling was negative, it was expected to see an increase in stress‐related responses prior to being handled. Stress‐related responses were hypothesised to include increased abnormal and inactive behaviours and decreased activity. Alternatively, if handling was not a stressful event for the snakes (or potentially positive), there should be minimal change in the above responses or a decrease in abnormal and inactive behaviours and increased activity.

## 2 Materials and Methods

Prior to the start of the research, animal ethics approval was granted by the University of Adelaide Animal Ethics Committee (S‐2025‐888) and exempted by the Department for Environment and Water (Y27586‐1).

### 2.1 Subjects and Housing

The observations for this project were conducted at Cleland Wildlife Park (South Australia, Australia) from late‐August to mid‐October. Three individual snakes across two species were observed: one woma python (*Aspidites ramsayi*) and two children's pythons (*Antaresia childreni*), as outlined in Table 1. An olive python (*Liasis olivaceus*) was also observed, although it was excluded from the results due to not being involved in any handling during the study duration. All snakes utilised in the study have been used by Cleland Wildlife Park in educational handling sessions regularly prior to the project.

**TABLE 1:** Information on the individual snakes used in the study.

Snake name	Bella	Helga	Nefti
Common name	Children's python	Children's python	Woma python
Species	*Antaresia childreni*	*Antaresia childreni*	*Aspidites ramsayi*
Snout‐to‐vent length (prior to the study; cm)	121	111	101
Weight (prior to the study; g)	563	702	493

All snakes were housed individually in separate enclosures within the park's education centre and cared for by the park's team. General zoo visitors did not have access to these enclosures, but school and other group visits did, with zoo staff present, during the hours of (10:00–16:30). Each enclosure was temperature‐controlled with both an infrared globe and underfloor heating, fitted with environmental enrichment, including hides, branches, and rocks, and each contained a water bowl. A UV light regulated by a timer in each enclosure provided appropriate ultraviolet light exposure and maintained a 12:12 day–night cycle. All enclosures consisted of walled sides, with a glass front for snake observation. Specifications for each enclosure are outlined in Table 2. Snakes were fed on a regular fortnightly schedule, with fresh water available ad libitum in each enclosure.

**TABLE 2:** Information on the enclosures used in the study.

Snake	Size of enclosure (cm)	Average max temp (C°)	Average min temp (C°)	Average max humidity (%)	Average min humidity (%)	Visual of enclosure
Bella	92 × 77 × 51	25.3	17.9	53	31	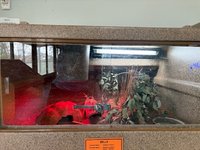
Helga	92 × 77 × 51	34.5	20.6	41	21	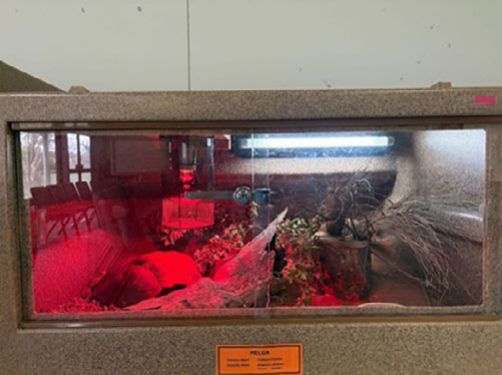
Nefti	149 × 77 × 51	27.6	21.6	53	43	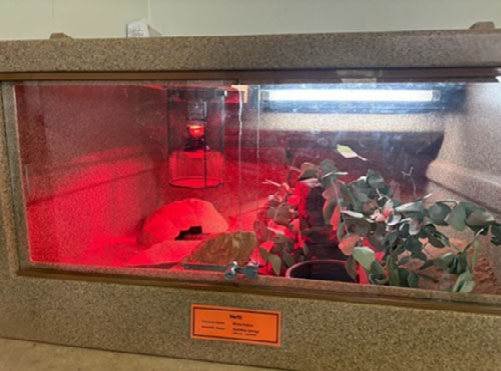

### 2.2 Materials

Materials included handheld tablets with the ZooMonitor (version 5.1.4) app (Wark et al., 2019), and three GardePro A60 Non‐Cellular Cameras to record time‐lapse videos. Additionally, tripods and zip‐ties were used to fix the cameras in position, and tape was used to mark the floor.

### 2.3 Handling Interactions

Each snake was involved in animal handling sessions led by trained staff. During these sessions, snakes were held by trained education officers, and visitors observed and could touch the caudal body with an open palm. Snakes were not handled if they showed signs of shedding or during and immediately following feed days. Interactions occurred on an irregular basis for approximately 10 min. A run sheet detailing the time, date, and snake involved during these interactions was maintained and later used to align interactions with behavioural data for analysis. During these sessions, the cameras were moved temporarily into storage to ensure that observers were blind to which snake was being utilised. Outside of these handling sessions, there was no visitor access to the education centre.

### 2.4 Data Collection

Observational data were obtained from video footage of each snake's enclosure. A video trap camera (GardePro A60 Non‐Cellular Camera) was positioned outside each enclosure using a tripod, ensuring the whole enclosure was captured. The cameras were set to record 10‐s clips at 5‐min intervals for a 10‐h period every day. The recording periods were from 08:00 to 18:00 between 22 August and 4 October 2025 and from 09:00 to 19:00 between 5 and 17 October 2025. Recordings were captured on both interaction and non‐interaction days to allow behavioural comparison. The SD cards and batteries from the cameras were replaced weekly to ensure continuous data collection.

### 2.5 Procedure

To assess the impact of handling on the subject's behaviour, two comparisons were conducted:

**Handling and Non‐Handling Days**: Comparisons made between observations on days where a snake was handled and days in which that individual was not handled.

**Pre‐ and Post‐Handling**: Comparisons were made between the immediate hours (see below) before (pre‐) and after (post‐) an individual snake was handled.

Behavioural data utilised in these comparisons were captured from 2‐h periods immediately before and after the cameras were stored for each handling session, as these were the closest behaviours observed to the handling events. Pre‐ and post‐handling comparisons used these 2‐h windows on days the snake was handled. Handling and non‐handling days comparisons combined these 2‐h windows on days the snakes were handled and were compared to equivalent time periods on non‐handling days, during which cameras were stored while another snake was being handled.

### 2.6 Data Coding

Video data were manually coded using a mutually exclusive ethogram developed specifically for this project (see Table 3), adapted from established reptile behavioural studies (Hanson et al. 2026). ZooMonitor was utilised to code the behaviour for the project. For each handling session and for each snake, footage captured in the 2 h prior and 2 h after was coded, as well as 4 h on a non‐handling day. For each 10‐s clip, one of the 16 behaviours was recorded as being observed at the 5‐s mark using focal instantaneous pinpoint sampling (Altmann 1974; Bateson and Martin 2021). The 16 behaviours were grouped into five behavioural classes: Active, Inactive, Abnormal, Out of sight, and Other, with each class having numerous behaviours. The “Other” behavioural class was included to allow the ethogram to be exhaustive. However, no instances of the behaviour were recorded throughout the project; therefore, it was excluded from all further analyses.

**TABLE 3:** Ethogram detailing snake behaviours, classes of behaviour, and definitions.

Behavioural class & behaviours (abbreviation)	Description
*Active*	
Locomotion in view (LoIV)	More than 25% of the body is in view and moving in any direction to a new location
Locomotion out of view (LoOV)	Less than 25% of the body is in view and moving in any direction to a new location
Climbing (Cl)	Animal is using branches and other furnishings in the enclosure to change elevation up or down
Investigative (In)	Head and neck moving about at a relaxed pace, exploring immediate surroundings, with or without tongue flicking
*Inactive*	
Coiled view (CV)	Non‐movement, with more than 25% of the body in view and more than 75% of the body assumes a spiral position, where the body is lapped at least three times
Stretched out (SO)	No movement with more than 25% of the body in view, stationary, and not in coiled position
*Abnormal*	
Freezing (Fr)	Head and neck are elevated, the body and head are stationary, and no tongue flicking is occurring
Window strike (WS)	Mock strikes towards the observer; the head is elevated off the ground, recoils into an “S”/”Z” shape, then lunges forward, a quick movement and hitting the glass of the enclosure. The snake begins more than 10 cm away from the glass and connects with the glass in less than 2 s
Boundary contact (BC)	Head is pushing, “surfing”, climbing, or digging at the walls/windows of the enclosure
Rapid body movement (RBM)	Abnormal movement that appears “jerky”. Can include the whole body or just the head and neck
Tail flicking (TF)	Tail is moving from side to side. The rest of the body is stationary
*Out of sight*	
Obstructed view (OV)	Animal is viewable with difficulty or using objects within the enclosure to reduce visibility; not enough is viewable to ascribe any other behaviour
In hide (Hi)	Animal is in their purpose‐built hide
*Other*	
Yawning (Y)	Animal's body is stationary; mouth is agape and then closes
Drinking (Di)	Mouth in contact with water
Defecating (De)	Excreting waste
Other (Ot)	Animal is displaying behaviour not described

*Note*: Adapted from Hanson et al. (2026).

Across all snakes, there were a total of (1) 32 1‐h sessions for pre‐ and post‐handling observations and (2) 68 1‐h sessions for handling and non‐handling day observations.

Two observers (C.K. and C.W.) completed all observations, with both observers blind to the condition that was coded. Because only two observers and a limited number of variables were recorded, no interrater reliability was measured. Instead, both observers were trained to collect data via the ethogram by the other two authors (S.H. and E.J.F.), first via visual demonstration and video, then live at the facility.

### 2.7 Data Analysis

Data were downloaded from ZooMonitor and entered into Microsoft Excel, where the mean and standard error of the mean (SEM) were calculated for each individual snake and for all three snakes combined to provide a more comprehensive overview of behavioural patterns. Behaviour was divided into four functional classes (Active, Inactive, Abnormal, and Out of sight) to examine general trends in activity and visibility. The data from each observation session were converted to percentages from raw counts to account for differences in total observation numbers and allow for a standardised measure of average activity levels and behavioural composition.

Data were then imported into SigmaPlot, version 14.0 (Systat Software Inc., San Jose, CA, USA) to generate visual representations of the results. Graphs were generated to illustrate changes in time spent in each behavioural category between handling and non‐handling days and between pre‐ and post‐handling conditions. Friedman's two‐way ANOVA by ranks was then performed in SPSS, version 31.0 (IBM Co., Armonk, NY, USA) to account for the repeated measures design and non‐normal distribution of the data.

## 3 Results

**FIGURE 1:**
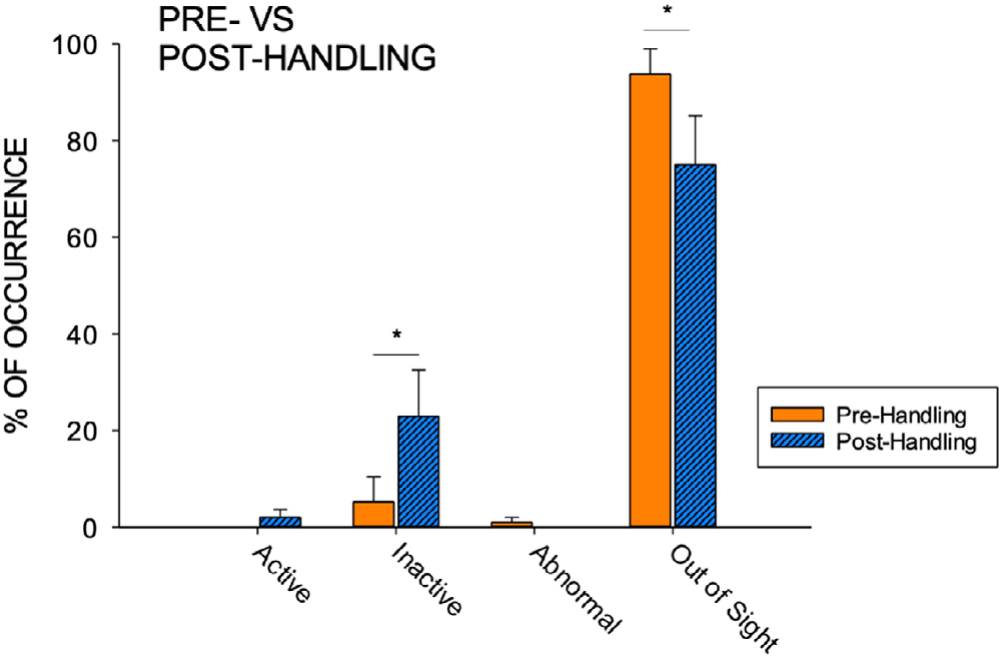
Combined mean percentage of behavioural occurrences pre‐ versus post‐handling, with solid lines representing significant differences (*p* < 0.05). Error bars represent the standard error of the mean (SEM).

A total of 192 behavioural observation intervals of three snakes were conducted pre‐ versus post‐handling sessions with staff using Friedman's two‐way ANOVA by ranks. This analysis allowed for the comparison of the frequency of each behaviour category (Active, Inactive, Abnormal, and Out of sight) across pre‐ and post‐handling conditions for all snakes. The results revealed that handling significantly affected the proportion of inactive and out of sight behaviours, while active and abnormal behaviours stayed relatively similar pre‐ versus post‐handling (see Figure 1). The proportion of inactive behaviour increased following handling (*χ* ^2^(1) = 6.00, *p* = 0.014), while out of sight behaviour decreased (*χ* ^2^(1) = 6.00, *p* = 0.014). No statistical significance was detected in the differences for active (*χ* ^2^(1) = 2.00, *p* = 0.157) and abnormal (*χ* ^2^(1) = 1.00, *p* = 0.317) behaviours pre‐ or post‐handling. Descriptive mean ± SEM values for each behavioural category are presented in Table 4.

**TABLE 4:** Mean ± SEM percentage of behavioural occurrences pre‐ versus post‐handling.

Behaviour category	Pre‐handling, M ± SEM (%)	Post‐handling, M ± SEM (%)
Active	0.00 ± 0.00	2.08 ± 1.56
Inactive	5.21 ± 5.21	22.92 ± 9.70
Abnormal	1.04 ± 1.04	0.00 ± 0.00
Out of sight	93.75 ± 5.25	75.00 ± 10.09

**FIGURE 2:**
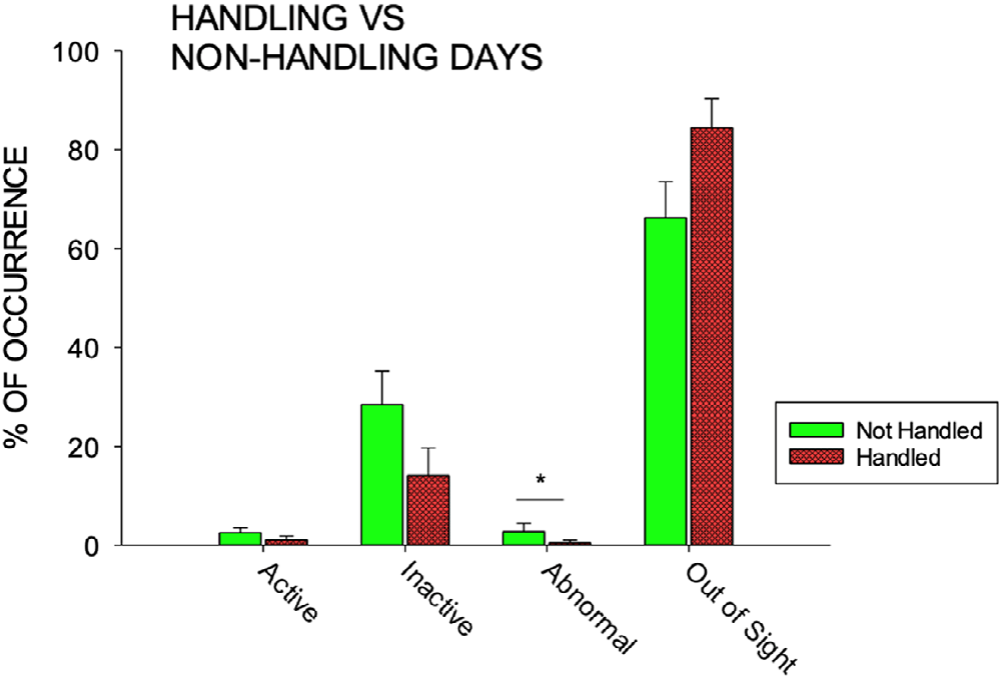
Combined mean percentage of behavioural occurrences on handling versus non‐handling days, with solid lines representing significant differences. Error bars represent the standard error of the mean (SEM).

The same statistical analysis was then used for the observation intervals of three snakes across handling versus non‐handling days using Friedman's two‐way ANOVA by ranks. This analysis allowed for the comparison of the frequency of each behaviour category (Active, Inactive, Abnormal, and Out of sight) across handling and non‐handling conditions for all snakes. The results of the analysis revealed that the proportion of abnormal behaviour showed a substantial decrease on handling days (*χ* ^2^(1) = 4.50, *p* = 0.034) (see Figure 2). However, handling did not significantly alter the proportion of active (*χ* ^2^(1) = 2.78, *p* = 0.096), inactive (*χ* ^2^(1) = 2.25, *p* = 0.134), or out of sight (*χ* ^2^(1) = 3.20, *p* = 0.074) behaviours.

## 4 Discussion

### 4.1 General Findings

Following handling events (i.e., post‐handling), snakes showed significantly increased inactive behaviour and decreased out of sight behaviour, suggesting they were more visible and resting in view after handling. Additionally, abnormal behaviours were significantly lower on handling days compared to non‐handling days. Taken together, these findings indicate that handling had little to no negative welfare impact for the snakes. Similar neutral and potentially positive effects have been reported in other zoo species exposed to human interaction or visitor presence, where such interactions can act as environmental enrichment (Claxton 2011; Hosey, 2000; Nimon and Dalziel, 1992; Fernandez et al. 2009, 2021).

All snakes used in this study had been handled regularly in Cleland Wildlife Parks' education sessions. The non‐negative welfare outcomes observed as a result of handling may be attributed to these animals habituating to these interactions; habituation is more broadly defined as decreased responding after repeated or continued presentation of a stimulus (Bouton 2007; Domjan 2010). Hanson et al. (2026) noted that snakes may habituate to AVIs, and other authors have found that zoo non‐primates may be enriched by them (Fernandez et al. 2009; Lin et al. 2025; Sherwen and Hemsworth, 2019; Williams et al. 2021). However, as these studies did not examine direct AVIs, comparisons should be interpreted with caution. Nonetheless, consideration of habituation to handling should be made for the snakes in this study, as the results may not generalise to snakes not regularly handled.

Abnormal behaviours observed in this study could include freezing, boundary contact, window striking, rapid body movement, and tail flicking. According to Denommé and Tattersall (2025), boundary contact behaviours are biased towards known escape routes within enclosures and are associated with escape motivations. Boundary contacts were the most frequently observed abnormal behaviour among the subjects, and a near‐significant decrease in abnormal behaviours was recorded on handling days. This trend may indicate that handling was associated with reduced escape‐motivated behaviours in the snakes observed. Overall, the observed reduction in total abnormal behaviours suggests that handling may not negatively impact welfare in habituated individuals and could function as a neutral or potentially enriching event under these conditions. However, as noted above, caution should be taken in the interpretation and generalisation of these results, as this finding may be a direct outcome of habituation to handling for these snakes.

Although AVIs involving reptiles, including snakes, remain largely unstudied, evidence from the few existing studies, including those on tuataras and tortoises, shows that direct handling can negatively impact welfare (Freeland et al. 2020; van Heerbeek et al. 2022). These conclusions contradict the findings in this study that handling had a neutral or non‐negative impact on the snakes’ welfare. However, a study by Fulkerson (2025) found that ambassador snakes displayed mostly positive behaviour during educational programmes, while also showing increased exposure, activity, and undesired behaviours after programmes, suggesting that post‐handling activity may reflect arousal or disruption rather than a purely positive welfare response. The differences in this and other reptile‐based handling studies may also reflect species‐typical differences between snakes and other reptiles, handling protocols and experience, housing and husbandry conditions, and methodology used for the noted studies. Greater welfare assessment research for reptiles, including handling studies, should provide greater insight into these considerations.

### 4.2 Limitations

The small sample size (*n* = 3) is reflective of the nature of our study, as zoo welfare research often focuses on individuals and smaller sample sizes (Goulart et al. 2009). Two species were represented, each with low individual counts (*Antaresia childreni*, *n* = 2; *Aspidites ramsayi*, *n* = 1). Nonetheless, although statistically significant results were obtained, the small total sample size, low number of individuals per species, and inclusion of only two species limit the ability to make species‐ or individual‐level conclusions, reduce statistical power, and limit the generalisability of the findings to other snakes or zoo‐handling programmes.

Observational data were collected during daytime hours, from 08:00 to 18:00 between 22 August and 4 October 2025, and from 09:00 to 19:00 between 5 and 17 October 2025. The activity patterns of the species studied are both nocturnal and diurnal (Hosking and Martinic 2020; Read et al. 2011). Therefore, collection of data within the outlined hours may have missed key behaviours. In addition, this study relied exclusively on a behavioural ethogram to assess animal welfare. Incorporating additional measures, such as physiological indicators and enclosure use, would strengthen welfare assessment, as welfare implications in snakes and other species may not always be reflected through behaviour alone (Cannon et al. 2002; Mellor et al. 2020).

Finally, the snakes were housed under artificially controlled conditions, notably temperature. During handling procedures, individuals were removed from their home enclosures and exposed to different ambient conditions. Behavioural activity in snakes is strongly influenced by temperature and lighting, with thermoregulatory behaviour and activity level occurring in response to suboptimal temperatures (Warwick et al. 2013). Consequently, the temperature and lighting discrepancy between the enclosure and handling environments may have confounded the interpretation of behavioural responses to handling.

### 4.3 Future Directions

This study highlights that captive snake welfare is an under‐researched field that requires more attention, particularly with interactive AVIs being so prevalent (D'Cruze et al. 2019). Current snake welfare assessments are largely limited to behavioural ethograms, thus making determining the valence of welfare assessments challenging (Hanson et al. 2026). Therefore, greater inclusion of additional measures, including the use of physiological assessment of potential stress and overall welfare (Barnett and Hemsworth 1990; Silvestre 2014), as well as the use of other measures such as behavioural diversity and enclosure use variability (Miller et al. 2020; Ross et al. 2009), would aid in better understanding captive snake needs. Due to limited research, many husbandry practices and welfare interpretations rely on anecdotal observations and assumptions, often referred to as “folklore husbandry” (Arbuckle 2013; Hanson et al. 2025; Warwick 2023). As a result of these limitations, there is a lack of consensus on which welfare indicators are meaningful or reliable for snakes.

Further research should aim to establish validated welfare indicators that reflect both positive and negative states, combining behavioural, physiological, and environmental measures to achieve a more complete understanding of snake welfare. Expanding studies to include more species, larger sample sizes, and across facilities would aid in consolidating and generalising findings and improve statistical power. Additionally, future work should address the substantial knowledge gaps surrounding the biological and environmental parameters that shape reptile welfare assessments (Mendyk and Warwick 2023). Addressing these foundational gaps will provide the necessary framework to move from assumption‐based welfare practices towards evidence‐based management for captive snakes.

## 5 Conclusion

This study provides preliminary evidence that regular, staff‐led handling was associated with increased visibility and reduced abnormal behaviours, suggesting that when appropriately conducted, handling can result in a reduction in negative welfare outcomes. However, methodological limitations constrain the generalisability of the findings, particularly as they related to potential habituated responses by the snakes to the handling procedures. Overall, this research contributes to a growing body of scientific evidence that supports the importance of evaluating reptile welfare rather than relying on assumptions or traditional practices. Further research with larger, more diverse samples and additional welfare measures is needed to better understand the effects of handling and AVIs on snake well‐being.

## Author Contributions

**Chelsea Kennedy**: investigation, writing – original draft, formal analysis, data curation, validation. **Sara L. Hanson**: writing – review and editing, project administration, supervision, validation, formal analysis, conceptualization. **Connor Walker**: writing – original draft, validation, formal analysis. **Eduardo J. Fernandez**: conceptualization, methodology, writing – review and editing, project administration, data curation, supervision, formal analysis.

## Funding

The authors have nothing to report.

## Ethics Statement

This study was approved by the University of Adelaide Animal Ethics Committee (S‐2025‐888) and exempted by the Wildlife Ethics Committee of the Department for Environment and Water (Y27586‐1).

## Conflicts of Interest

The authors declare no conflicts of interest.
